# Cardiac Involvement in Sarcomatoid Renal Cell Carcinoma

**DOI:** 10.7759/cureus.14076

**Published:** 2021-03-24

**Authors:** Amr Mohamed, Maha Mohamed

**Affiliations:** 1 Internal Medicine, Rochester Regional Health, Rochester, USA; 2 Pathology, Ain Shams University, Cairo, EGY

**Keywords:** renal cell metastasis, cardiac tumours

## Abstract

Pulmonary emboli (PE) in malignancy are usually related to hypercoagulability; however, in rare situations, direct tumor emboli are the etiology of pulmonary embolism. We present here a case of a 68-year-old male with known stage IV sarcomatoid renal cell carcinoma who came to the emergency department (ED) complaining of shortness of breath. A CT scan was done that showed bilateral segmental PE and a cardiac mass in the right ventricle that was consistent with known renal cell carcinoma. He was started on anticoagulation with low molecular weight heparin; six months later, he presented to the ED with worsening shortness of breath, and repeat CT showed an increased clot burden in the pulmonary arteries with new right ventricular (RV) strain on CT despite anticoagulation. A decision was made to go for cardiac MRI to check if the cardiac metastasis could be removed as it was thought to be the source of embolization. Cardiac MRI showed cardiac metastasis near the RV outflow tract. Unfortunately, before surgical planning, he was admitted with fatal intra-abdominal bleeding from the tumor, and passed away despite angiographic embolization and resuscitation. PE from renal cell carcinoma are sometimes tumor emboli rather than related to hypercoagulability, and this sometimes needs a different intervention compared to ordinary pulmonary embolism management, as shown in this case.

## Introduction

Primary cardiac tumors are rare [1]; on the other hand, metastatic cardiac tumors are about 20 times more common. The presentation is also variable, as they can be symptomatic, asymptomatic, or incidentally found [2]. Pulmonary emboli (PE) related to any tumor are either associated with hypercoagulability or rarely direct tumor emboli. Here, we are presenting a case of pulmonary tumor emboli related to renal cell carcinoma.

## Case presentation

**Figure 1:**
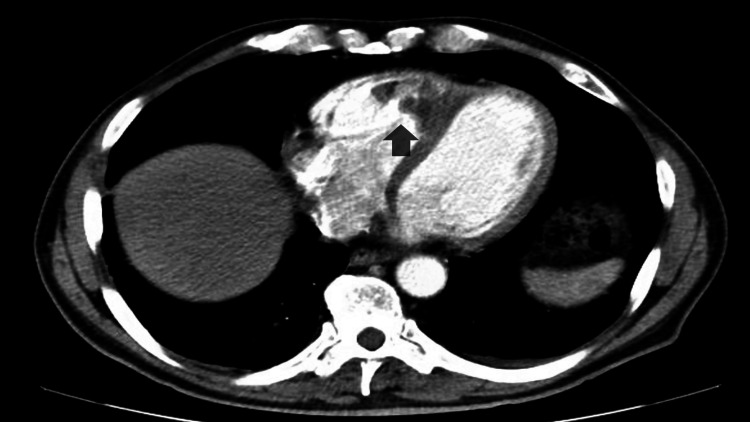
CT scan of the chest with IV contrast showing lobulated mass within the right ventricle (the arrow is pointing toward the intra-cardiac tumor)

A 68-year-old male with known stage IV sarcomatoid renal cell carcinoma presented to the emergency department (ED) complaining of shortness of breath; his vital signs were unremarkable with stable oxygen saturation, and his physical examination had been normal. Because of the high suspicion of pulmonary embolism related to his risk factors, a CT scan of the chest with IV contrast was done, which had shown bilateral segmental and sub-segmental PE and a cardiac mass in the right ventricle that is consistent with known renal cell carcinoma as shown in Figure 1. There was no right ventricular (RV) strain on CT or transthoracic echo, and troponin and B-type natriuretic peptide (BNP) had been normal. Given that he was stable, a decision was made to start him on therapeutic anticoagulation with low molecular weight heparin with close follow-up.

Six months later, he presented to the ED with worsening shortness of breath. A CT scan was repeated at that time, showing an increased clot burden in the pulmonary arteries at segmental and sub-segmental levels but now with evidence of new RV strain on CT despite him being on a therapeutic dose of anticoagulation. It was thought that this was not related to hypercoagulability, but instead, these were tumor emboli related to his cardiac metastasis.

**Figure 2:**
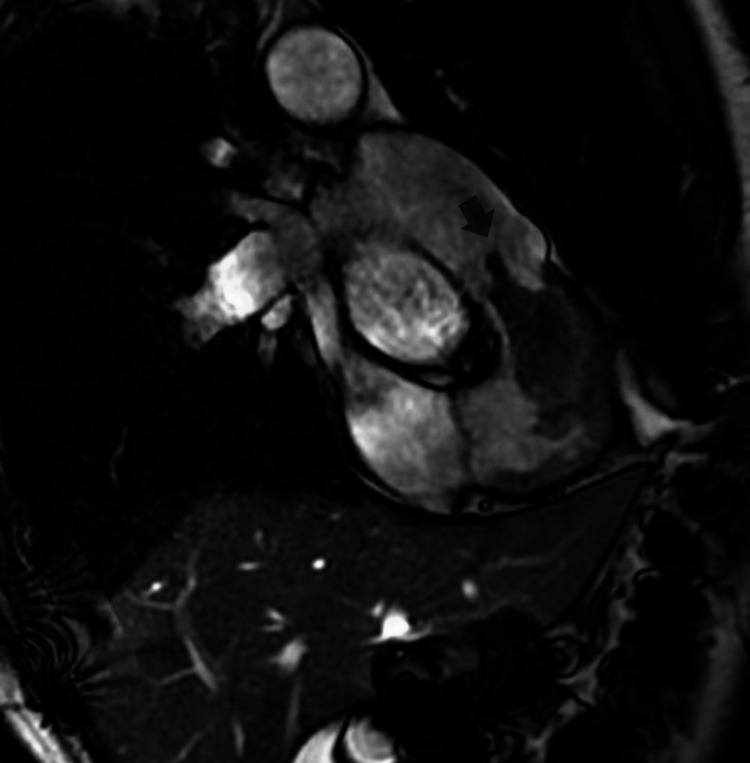
Cardiac MRI showing a large right ventricular mass attached to the endocardium on the RV septal aspect and the anterior aspect of the RVOT (the arrow is pointing toward the intra-cardiac tumor) RV, right ventricular; RVOT, RV outflow tract

**Figure 3:**
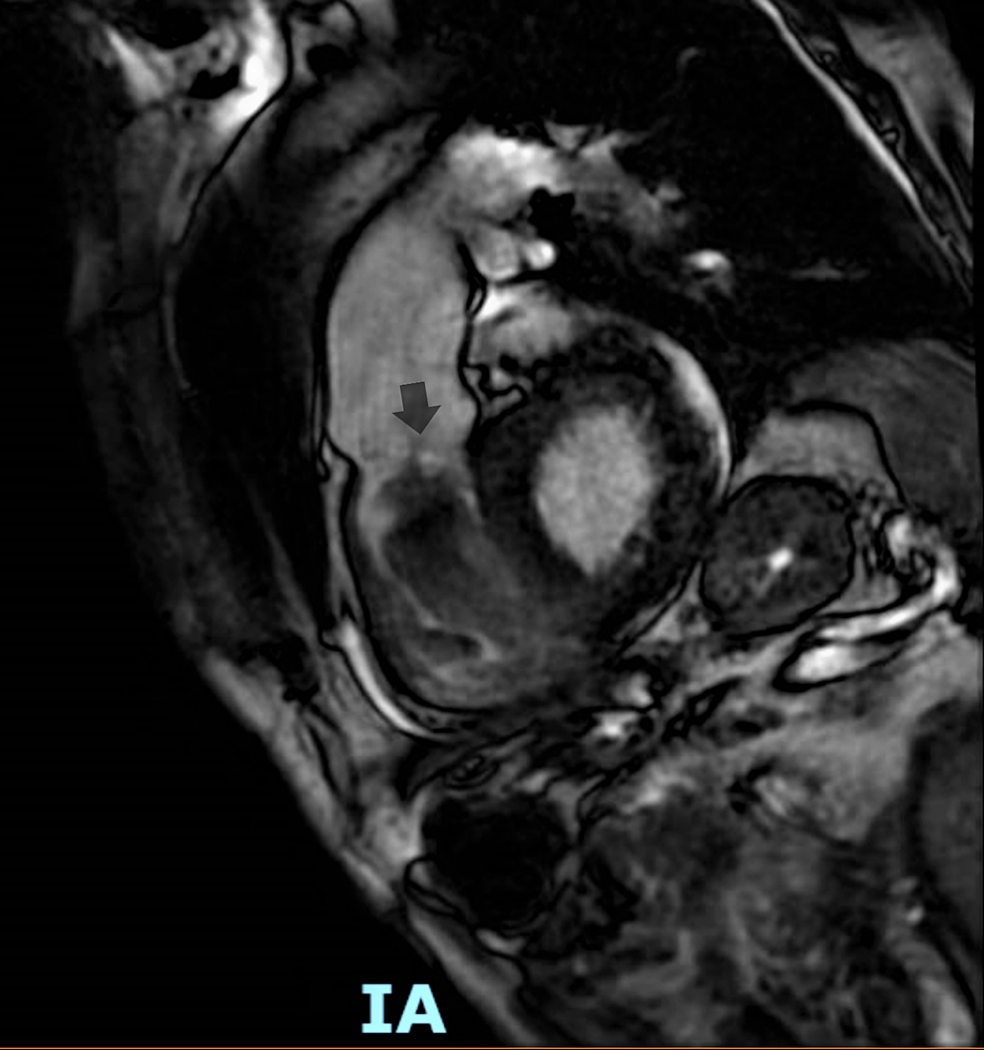
Cardiac MRI showing a large right ventricular mass attached to the endocardium on the RV septal aspect and the anterior aspect of the RVOT (the arrow is pointing toward the intra-cardiac tumor) RV, right ventricular; RVOT, RV outflow tract

**Figure 4:**
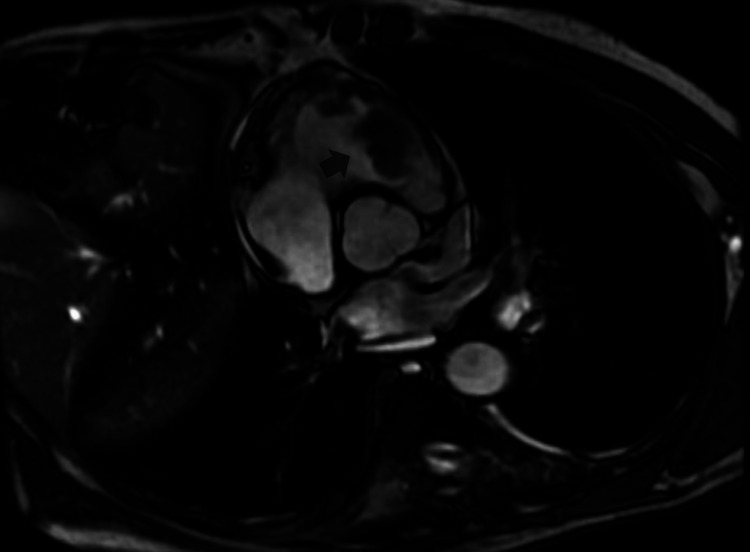
Cardiac MRI showing a large right ventricular mass attached to the endocardium on the RV septal aspect and the anterior aspect of the RVOT (the arrow is pointing toward the intra-cardiac tumor) RV, right ventricular; RVOT, RV outflow tract

Cardiothoracic surgery was consulted, and as recommended, cardiac MRI was done to see if the cardiac metastasis could be removed surgically. Cardiac MRI showed a large right ventricular mass attached to the endocardium on the RV septal aspect and the anterior aspect of the RV outflow tract (RVOT); the mass was highly mobile and prolapsing into the pulmonary artery in real-time view (Figures 2-4).

Surgical option was considered because the mass was near the RVOT and was prolapsing into the pulmonary arteries. It was feared that massive tumor embolization could happen later on, leading to a high-risk pulmonary embolism and cardiac arrest. The patient also had good functional status despite having stage IV renal cell carcinoma.

**Figure 5:**
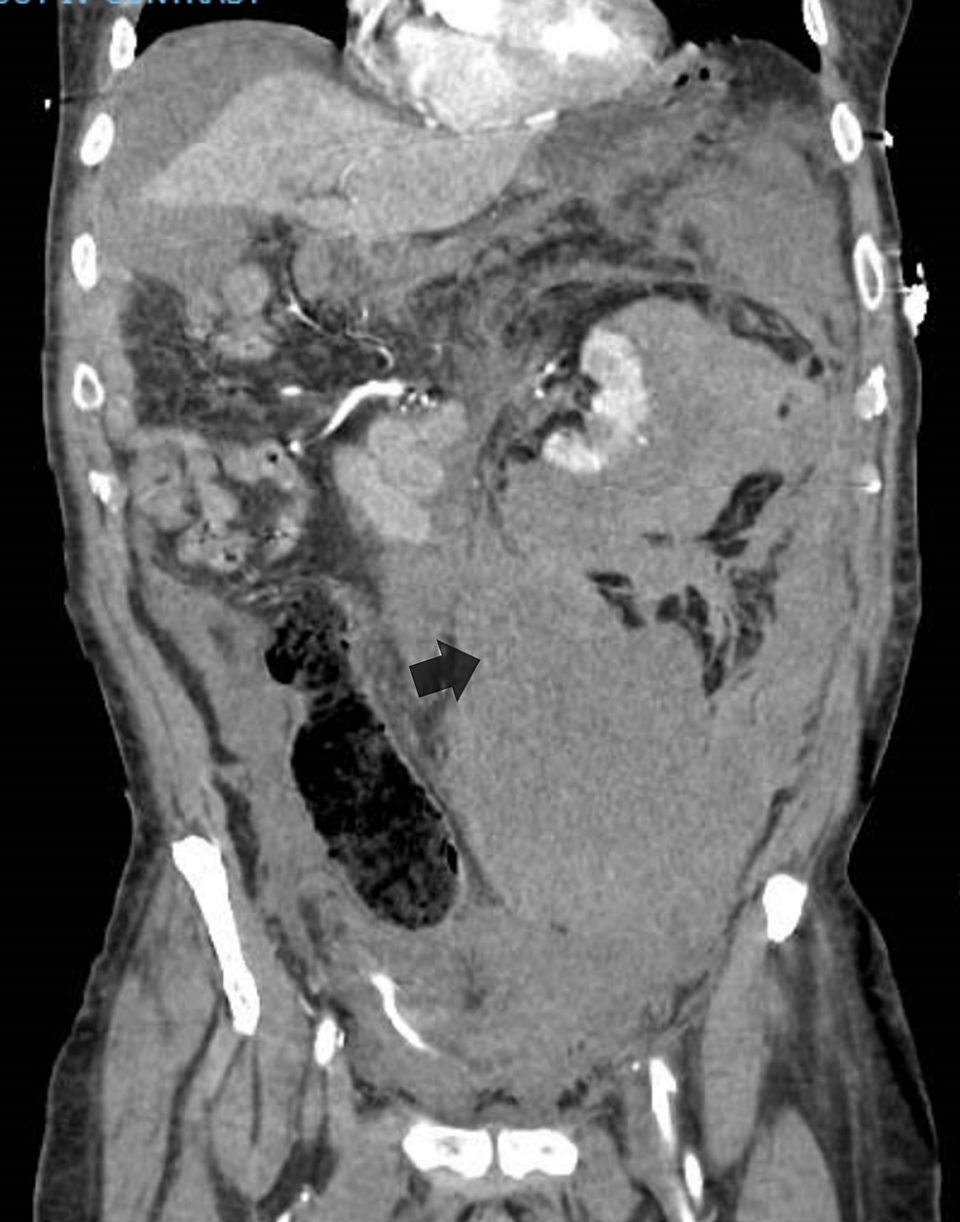
CT scan of the abdomen and pelvis showing massive intra-abdominal bleeding related to renal cell carcinoma (the arrow is pointing toward the intra-abdominal bleeding)

Unfortunately, before surgical planning, he was admitted with fatal intra-abdominal bleeding from the tumor, as shown in Figure 5, and passed away despite angiographic embolization and resuscitation.

## Discussion

Two things need to be discussed here: first, cardiac metastasectomy, and second, the idea of pulmonary tumor emboli. When reviewing the literature, to our knowledge, no strong evidence supporting cardiac metastasectomy is found. Most of the literature evidence is case reports for various types of tumors with a variable success rate. In the case of renal cell carcinoma, it is very challenging and is primarily a palliative option [3].

In our patient, we decided to go for cardiac metastasectomy given an increased pulmonary clot burden on interval CT despite full-dose anticoagulation and because the RVOT mass was highly mobile and prolapsing into the pulmonary artery, contributing to a very high risk of massive pulmonary embolization. We were afraid that the next phase would be massive PE related to this mass. Surgery was not done because the patient died from another etiology.

The concept of cardiac metastasectomy needs to be further studied; the main obstacle in further studies is the assessment of patient candidacy for the surgery and the surgical risk for these patients with already poor life expectancy and multiple comorbidities. Other barriers to surgery are tumor location and the extent of ventricular wall invasion [4]. Other treatment options include chemotherapy, immunotherapy, or local radiation depending on the sensitivity pattern, but each comes with its own side effects [4].

Secondly, when a patient with malignancy presents with pulmonary embolism, this is often related to hypercoagulability. However, in a small subset, this is pulmonary tumor emboli. The management is essentially the same in addition to focusing on treating the underlying tumor, and cardiac metastasectomy is usually done as a last resort, mainly as palliative treatment with many limitations.

## Conclusions

Pulmonary emboli from renal cell carcinoma are sometimes tumor emboli rather than related to hypercoagulability, and this sometimes needs a different intervention compared to ordinary pulmonary embolism management, as shown in this case.

## References

[1] Reynen K (1996). Frequency of primary tumors of the heart. Am J Cardiol.

[2] Lam KY, Dickens P, Chan AC (1993). Tumors of the heart. A 20-year experience with a review of 12,485 consecutive autopsies. Arch Pathol Lab Med.

[3] Aburto J, Bruckner BA, Blackmon SH, Beyer EA, Reardon MJ (2009). Renal cell carcinoma, metastatic to the left ventricle. Tex Heart Inst J.

[4] Zhang B, Malouf J, Young P, Kohli M, Dronca R (2013). Cardiac metastasis in renal cell carcinoma without vena cava or atrial involvement: an unusual presentation of metastatic disease. Rare Tumors.

